# Outbreak investigation of CRAB at an acute-care hospital ICU during the COVID-19 pandemic–Chicago, Illinois, March 2020–September 2021

**DOI:** 10.1017/ash.2022.215

**Published:** 2022-05-16

**Authors:** Hira Adil, Kelly Walblay, Shelby Daniel-Wayman, Massimo Pacilli, Shannon Xydis, Christine Pate, Ann Valley, Stephanie Black

## Abstract

**Background:** Carbapenem-resistant *Acinetobacter baumannii* (CRAB) is primarily associated with hospital-acquired infections and is an urgent public health threat due to its ability to contaminate the environment and cause severe disease. In 2019, Illinois began pilot surveillance for CRAB requiring select laboratories to submit specimens for molecular characterization. On July 17, 2020, the Chicago Department of Public Health (CDPH) was notified of an increase in CRAB infections in a 20-bed ICU at an acute-care hospital in Chicago (hospital A) during the initial COVID-19 surge. We summarize the outbreak investigation findings and infection control recommendations. **Methods:** Clinical cultures were collected from patients in hospital A, and CRAB-positive isolates were sent to the Wisconsin State Laboratory of Hygiene for mechanism of resistance and antibiotic susceptibility testing. On-site assessments and remote follow-ups were conducted by CDPH infection preventionists to evaluate infection control practices including environmental cleaning, hand hygiene compliance, and use of personal protective equipment (PPE). The Illinois Department of Public Health and CDPH summarized the testing results, facilitated a containment response, and provided recommendations for infection control. **Results:** From March 18, 2020, to September 30, 2021, 56 patients with CRAB infections were identified from hospital A, and 33 (59%) of these cases were pan-nonsusceptible. Most specimen sources were sputum (n = 30, 54%), followed by blood (n = 13, 23%), urine (n = 6, 11%) and other (n = 7, 13%). Among isolates with mechanism testing (n = 54), 45 (83%) were positive for OXA-24/40 and 9 (17%) were positive for OXA-23. Of the CRAB-positive patients, 28 (50%) were previously positive for SARS-CoV-2. To date, 25 of these patients (45%) have been discharged and 31 (55%) have died. Two onsite visits and 7 remote-assistance sessions were conducted as part of the investigation. In response to increased COVID-19 hospitalizations, hospital A moved to crisis-capacity PPE use and encountered staffing shortages, which led to compromised infection control measures. Cleaning agents (Quat disinfectant cleaner) were also found to be ineffective against CRAB and required long contact times. **Conclusions:** In response to the CRAB outbreak at hospital A, CDPH recommended that the hospital stop crisis-capacity protocols for PPE, conduct admission screening and point-prevalence testing for CRAB, implement a hand hygiene campaign, and use an EPA-registered List K product for environmental cleaning. These recommendations were implemented in May 2021, and no CRAB cases have been reported since July 2021. To reduce CRAB transmission during the pandemic, facility leadership must commit resources to educate staff on effective infection control practices including conventional use of PPE, appropriate cleaning agents, and improved hand hygiene.

**Funding:** None

**Disclosures:** None

